# Reference gene validation for gene expression normalization in canine osteosarcoma: a geNorm algorithm approach

**DOI:** 10.1186/s12917-017-1281-3

**Published:** 2017-11-25

**Authors:** Gayathri Thevi Selvarajah, Floor A. S. Bonestroo, Elpetra P. M. Timmermans Sprang, Jolle Kirpensteijn, Jan A. Mol

**Affiliations:** 1 Department of Clinical Sciences of Companion Animals, Faculty of Veterinary Medicine, University of Utrecht, Yalelaan 104, 3584 CM Utrecht, The Netherlands; 2 Department of Veterinary Clinical Studies, Faculty of Veterinary Medicine, University Putra Malaysia, UPM, 43400 Serdang, Malaysia

**Keywords:** Quantitative real-time PCR, Osteosarcoma, Bone tumor, Dog, Reference genes

## Abstract

**Background:**

Quantitative PCR (qPCR) is a common method for quantifying mRNA expression. Given the heterogeneity present in tumor tissues, it is crucial to normalize target mRNA expression data using appropriate reference genes that are stably expressed under a variety of pathological and experimental conditions. No studies have validated specific reference genes in canine osteosarcoma (OS). Previous gene expression studies involving canine OS have used one or two reference genes to normalize gene expression. This study aimed to validate a panel of reference genes commonly used for normalization of canine OS gene expression data using the geNorm algorithm. qPCR analysis of nine canine reference genes was performed on 40 snap-frozen primary OS tumors and seven cell lines.

**Results:**

Tumors with a variety of clinical and pathological characteristics were selected. Gene expression stability and the optimal number of reference genes for gene expression normalization were calculated. *RPS5* and *HNRNPH* were highly stable among OS cell lines, while *RPS5* and *RPS19* were the best combination for primary tumors. Pairwise variation analysis recommended four and two reference genes for optimal normalization of the expression data of canine OS tumors and cell lines, respectively.

**Conclusions:**

Appropriate combinations of reference genes are recommended to normalize mRNA levels in canine OS tumors and cell lines to facilitate standardized and reliable quantification of target gene expression, which is essential for investigating key genes involved in canine OS metastasis and for comparative biomarker discovery.

## Background

Osteosarcoma (OS) is the primary malignant bone tumor in dogs. Apart from having complex metastatic characteristics, OS has been observed to have a complex histopathology that develops due to predominantly osteoblastic cell differentiation as well as a mixture of fibroblastic and chondroblastic cell differentiation, with varying degrees of necrosis and tumor matrix present within a tumor [1, 2]. Gene expression studies in canine OS are valuable, as dogs develop OS spontaneously and have many common clinical and molecular characteristics that are invaluable resources for biomarker discovery and offer translational opportunities [3, 4]. Furthermore, publication of the canine genome along with the advent of quantitative real-time PCR (qPCR) and other high-throughput technologies have enabled studies of key genes involved in OS metastasis and disease progression.

qPCR is a sensitive method for quantifying mRNA gene transcripts; the two most popular real-time assays use SYBR® green fluorescent dye and the Taqman® probe. Many reports have demonstrated the importance of studying gene expression at the mRNA transcription level using snap-frozen tissues, micro-dissected tumors from paraffin-embedded blocks [5], cellular content from fine needle aspirates of primary tumors, and various cell culture models. The quantification of gene expression using the qPCR method requires appropriate standardization from initial tissue sampling, RNA extraction protocols, cDNA synthesis, assay characteristics, and reference gene validation [6, 7]. Furthermore, it is important to incorporate internal standards such as reference genes to normalize mRNA expression levels between different samples to precisely compare mRNA transcription levels. Ideally, a reference gene should be stably expressed in tissues or cells regardless of the histology, pathological condition, or cellular physiological-metabolic state.

Reference gene expression validation studies have been conducted in several types of normal, diseased, and tumor canine tissues [8, 9]. These studies suggested that stably expressed genes can differ according to the tissue origin and disease condition, particularly in cancer. Most gene expression studies examining canine OS have included one or two reference genes as the internal control for data normalization [4, 10–14]. Given the biological and pathological diversity of OS tumors, it is crucial to determine the stability of reference genes and their suitability for normalization to accurately quantify gene expression data. Thus, in the present study, the mRNA expression of nine commonly used canine reference genes was quantified using the SYBR® green fluorescent dye qPCR assay with canine OS snap-frozen tissues and cell lines. The geNorm algorithm approach was utilized to determine the reference gene(s) showing stable expression for normalization of canine OS mRNA expression data.

## Methods

All procedures were approved by the University of Utrecht, Netherlands ethical committee, as required under Dutch legislation. Naturally developed bone tumors were obtained from privately owned euthanized animals or obtained through a routine medical treatment for cancer (surgical resection of tumors) at the Department of Clinical Sciences of Companion Animals (University Clinic for Companion Animals) in Utrecht, The Netherlands. No experimental animals were used for the sole purpose of this study.

### Tissue specimens and clinical-pathological data

Of the dogs with OS clinically diagnosed at the University Clinic for Companion Animals in Utrecht, The Netherlands, 40 with histologically confirmed primary tumors were selected for this study. Tissues from these samples were harvested under sterile conditions during surgery (amputation/marginal resection/total resection), snap-frozen in liquid nitrogen, and stored at −70 °C. Histopathology diagnosis and grading [2] were performed by a certified veterinary pathologist. These 40 tumors were selected after screening from 60 OS tumors randomly selected from the snap-frozen tumor archive at the Department of Clinical Sciences of Companion Animals, University of Utrecht; first based on RNA quantity (minimum 100 ng/μL in 30 μL) and followed by RNA quality (RIN > 6.5). The samples that didn’t qualify these two stages of screening were not included in this study. The medical records of the selected 40 tumors were reviewed retrospectively.

### Cell lines and culture conditions

Seven well-characterized canine OS cell lines were used in this study. The cell lines COS31 [15], HMPOS [16], and POS [17] were obtained through a collaboration with the University of Florida, USA; KOS-001, KOS-002, KOS-003 and KOS-004 were kindly gifted by the National Cancer Institute, NIH, Bethesda, MD, USA. All cell lines tested negative for mycoplasma using a myco-sensor qPCR assay kit according to the manufacturer’s protocol (Agilent Technologies, CA, USA). Cells were maintained in a sub-confluent monolayer in DMEM supplemented with 10% fetal bovine serum (Invitrogen, CA, USA) at 37 °C in a humidified atmosphere with 5% CO_2_.

### RNA isolation and cDNA synthesis

RNA in snap-frozen OS tumor materials was isolated as described previously [3, 18]. Briefly, frozen bone tumor materials were ground to form bone powder, which was subjected to RNA isolation protocols. For cells grown in culture, 1 mL of RLT lysis buffer (Qiagen, Germany) was used to lyse 75–90% confluent cells grown in 75 mL flasks, following a single wash of the cells with Hank’s Balance Salt Solution (PAA Laboratories, GmbH, UK). These three samples were collected from three independent passages in culture. RNA was isolated and cDNA synthesis done independently for the three samples and not pooled together. The three samples were considered as three independent biological replicates from each cell line. In addition to that, for qPCR assay, each of these biological replicate was assessed for gene expression in duplicate (technical replicate) using qPCR assays**.** RNA isolation and purification was performed using the RNeasy mini kit according to the manufacturer’s protocol (Qiagen). The RNA samples were treated with the Qiagen RNase-free DNase kit (DNase-I) and eluted in purified water. Total RNA was quantified using the Nanodrop ND-1000 spectrophotometer (Isogen Lifesciences, The Netherlands). RNA quality was evaluated using the Agilent 2100 Bioanalyzer (Agilent Technologies). The cDNA was synthesized using 0.5 μg total RNA into a total reaction volume of 20 μL from each sample using the iScript kit cDNA Synthesis Kit according to the manufacturer’s protocol (Bio-Rad, CA, USA).

### Quantitative real-time PCR

Primers were designed and qPCR products were sequenced for specificity as previously described [19, 20]. cDNA samples from both cell lines and tumors were diluted by two-fold, pooled, and diluted with purified water in a four-fold serial dilution to assess the amplification efficiency of each gene. The remaining cDNA samples were diluted by two-fold and 2 μL was used as a template to measure the gene expression in technical duplicates. qPCR was conducted on separate plates for the OS cell lines from the primary tumors using the SYBR® green fluorescent dye method. Initial screening for genomic DNA contamination was performed on all samples using a non-reversed-transcribed RNA template. qPCR was performed on a MyiQ™ quantitative real-time PCR machine (Bio-Rad). Reactions were conducted in duplicate, involving two-step reaction protocols, except for HPRT which involved a three-step reaction protocol, for up to 40 qPCR cycles [19, 20].

### Data analysis

Individual reaction data were corrected for qPCR efficiencies and analyzed using IQ5 software (Bio-Rad). A box-plot was generated from the absolute qPCR cycle threshold (Cq) values [6] referring to the RNA transcription of the tested reference genes in OS tissues and cell lines using the statistical software SPSS version 16.0 (SPSS, Inc., Chicago, IL, USA). Cases with values between 1.5 and 3.0 box length, from the upper or lower edges of the box, are presented as outliers and indicated by a dark dot. The expression stability of each reference gene in tumors and cell lines was calculated independently, and their average values were recalculated using step-wise exclusion and pairwise variation analyses, all of which were analyzed using geNorm (version 3.5) software [21]. GeNorm calculates the stability of expression (M) of one gene based on the average pairwise variation between all studied reference genes. The pairwise variation (V) value illustrates the variation generated by incorporating various numbers of reference genes for normalization based on individual absolute (M) values. A lower V value indicates lower variation between the selected combinations of reference genes. Stepwise elimination of the least stable gene reveals the two most stable genes.

## Results

### Canine OS samples and reference gene selection

Clinical and pathological data of 40 primary canine OS tissues from differently sized (medium to large) breeds used in this study are summarized in Table 1. The tissues were obtained upon amputation or tumor resection prior to the initiation of chemotherapy. These tumors consisted of mixed histopathology characteristics. Seven canine OS cell lines with varying characteristics, including morphology, cell proliferation, colony-forming abilities, migration, and apoptotic rates, were selected. Sub-confluent cells from 3 independent passages were lysed for RNA isolation, as representatives for biological replicates from each cell line. The reference genes selected for this study were previously described (e.g. *RPS19*, *HPRT*, *GAPDH*) [3, 18] and several putative reference genes that have not been used in OS studies, but were expressed in other canine tissues (e.g. *SRPR*, *HNRNPH*, *GUSB*, *RPL8*, *RPS5, B2M*) [19, 20]. These genes represent different functional groups, thus avoiding having a cluster of genes co-regulated in a specific cellular mechanism (Table 2).

**Table 1:** Characteristics of canine OS tissues (*n* = 40) used for this study

Parameter	*n*	%
Histological subtype^a^		
OB + FB	12	30
OB + TL	5	12.5
OB + CB + FB	7	17.5
OB + FB + TL	2	5
OB	14	35
Histological grade		
High	28	70
Medium-low	12	30
Necrosis		
< 50% (low)	12	30
> 50% (high)	28	70
Sex		
Female	14	35
Male	26	65
Neuter status		
Intact	22	55
Neutered	18	45
Location of primary tumor		
Extraskeletal	1	2.5
Femur	1	2.5
Humerus	8	20
Mandible/maxilla	3	7.5
Radius/ulna	14	35
Rib	2	5
Scapula	3	7.5
Tibia/fibula/metatarsus	8	20

^a^ *CB* chondroblastic, *FB* fibroblastic, *OB* osteoblastic, *TL* telangiectic

**Table 2:** Reference genes for canine OS and their cellular function(s)

Gene symbol	Name	Function
RPS5	Ribosomal protein S5	Ribosomal protein that is a component of the 40S subunit, belongs to the S7P family of ribosomal proteins
RPS19	Ribosomal protein S19	Ribosomal protein that is a component of the 40S subunit, belongs to the S19E family of ribosomal proteins
HPRT	Hypoxanthine guanine phosphoribosyl transferase	Purine metabolism, salvage of purines from degraded RNA
HNRNPH	Heterogeneous nuclear ribonucleoprotein H	RNA-binding protein that forms a complex with heterogeneous nuclear RNA (hnRNA). These proteins are associated with pre-mRNAs in the nucleus and appear to influence pre-mRNA processing and other aspects of mRNA metabolism and transport
RPL8	Ribosomal protein L8	Ribosomal protein that is a component of the 60S subunit which catalyzes protein synthesis
GAPDH	Glyceraldehyde-3-phosphate dehydrogenase	Enzyme in glycolysis and gluconeogenesis pathway
B2M	β-2-Microglobulin	Beta chain of MHC class I molecules
SRPR	Signal recognition particle receptor	Ensures, in conjunction with the signal recognition particle, the correct targeting of the nascent secretory proteins to the endoplasmic reticulum membrane system
GUSB	β-glucuronidase	Role in degradation of dermatan and keratin sulphates

### Pre-qPCR quality control measures and qPCR efficiencies

RNA quantity in tumors ranged from 173.0 to 2399.3 ng/μL, while the RNA quality of all samples was acceptable with a 260/280 ratio of 1.97–2.11. RNA integrity number (RIN) values were 9.5–10.0 for the cell lines and above 6.5 for the snap-frozen tumors. Primer sequences, product size, and optimal annealing temperature for each reference gene were previously verified [19, 20] and are summarized in Table 3. qPCR was performed in duplicate for each sample in which separate assays for cell lines and tumors were performed. Both the non-reverse transcribed template control samples were below the detection limits in every qPCR. qPCR efficiencies were between 91.1% and 103.1% for the cell lines and between 94.9% and 104.1% for the tumors. All qPCRs exhibited a single melting curve representing a specific product.

**Table 3:** Details of primers and qPCR conditions for the putative reference genes assessed in this study

Reference gene	Accession number	Forward primer 5′ to 3′	Reverse primer 5′ to 3′	Product length (bp)	T_a_ (°C)
RPS5	XM_533568	TCACTGGTGAGAACCCCCT	CCTGATTCACACGGCGTAG	141	62.5
RPS19	XM_533657	CCTTCCTCAAAAAGTCTGGG	GTTCTCATCGTAGGGAGCAAG	95	61
HPRT	AY_283372	AGCTTGCTGGTGAAAAGGAC	TTATAGTCAAGGGCATATCC	114	56
HNRNPH	XM_53857	CTCACTATGATCCACCACG	TAGCCTCCATAACCTCCAC	151	61.2
RPL8	XM_532360	CCATGAATCCTGTGGAGC	GTAGAGGGTTTGCCGATG	64	55
GAPDH	NM_001003142	TGTCCCCACCCCCAATGTATC	CTCCGATGCCTGCTTCACTACCTT	100	58
B2M	XM_535458	TCCTCATCCTCCTCGCT	TTCTCTGCTGGGTGTCG	85	61.2
SRPR	XM_03184	GCTTCAGGATCTGGACTGC	GTTCCCTTGGTAGCACTGG	81	61.2
GUSB	NM_001003191	AGACGTTCCAAGTACCCC	AGGTGTGGTGTAGAGGAGCAC	103	62

*T*_*a*_ annealing temperature, *bp* base pair

### Reference gene expression variation in OS tumors and cell lines

**Fig. 1:**
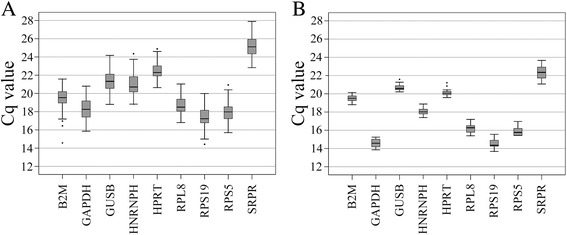
Box-plots demonstrating the absolute Cq values, 25%/75% percentiles, and outliers (indicated by dark dots) for mRNA transcription quantified for the putative reference genes in: **a** canine OS snap-frozen primary tumors and **b** for canine OS cell lines

Reference genes that were highly expressed in both OS tumors and cell lines, based on average Cq values, were *GAPDH*, followed by the ribosomal RNA genes *RPS19*, *RPS5*, and *RPL8*. *SRPR* showed the lowest expression. Although the absolute Cq range differed slightly between the tumor and cell line assays, a coherent expression pattern was observed. The expression range and average Cq values for each reference gene in OS tumors and cell lines are shown in Fig. 1.

### Expression stability of reference genes in canine OS tumors and cell lines

**Fig. 2:**
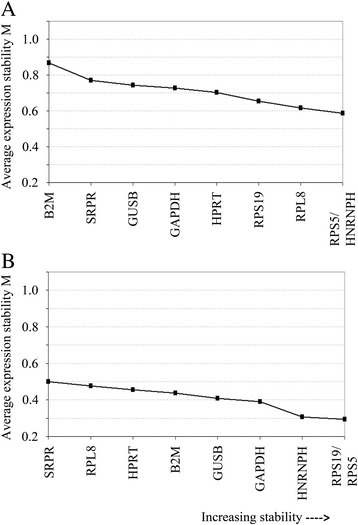
Expression plots generated by geNorm for **a** canine OS primary tumors and **b** canine OS cell lines for the average expression stability (M values) for the 9 tested genes upon step-wise exclusion method. Less stable genes were eliminated by the step-wise exclusion method and the average M value was re-calculated among the remaining candidate genes. The 2 most stable genes for OS primary tumors were *RPS5* and *HNRNPH*, while *RPS5* with *RPS19* were the most stable combination among cell lines

The average reference gene expression stability (M value) upon step-wise exclusion and pairwise variation (V value) were calculated using the geNorm algorithm approach for the tumors and cell lines individually. A higher absolute M value indicates lower expression stability and vice versa (Table 4). Among the reference genes tested for the canine OS cell lines, *HNRNPH* was the most stable gene with an M value of 0.420, while *SRPR* appeared to be the least stably expressed gene with an M value of 0.588, although all reference genes had acceptable M values. For OS tumors, absolute M values ranged from 0.790 for *RPS19* (most stable) to 1.210 for *B2M* (least stable) compared to the other reference genes. The average expression stabilities of the 9 tested reference genes among cell lines and tumors upon the stepwise exclusion algorithm are depicted in Fig. 2. *HNRNPH* and *RPS5* expression, together, showed the lowest variability for the cell lines, while *RPS19* and *RPS5* were the best combination for the tumors.

**Table 4:** Reference genes ranked based on their expression stability, M, in canine osteosarcoma primary tumors and cell lines

Primary tumors (tissues)		Cell lines	
Gene	M value	Gene	M value
RPS19	0.790	HNRNPH	0.420
RPS5	0.796	RPS5	0.423
HNRNPH	0.803	B2M	0.475
HPRT	0.808	RPS19	0.494
GUSB	0.816	GUSB	0.508
GAPDH	0.835	HPRT	0.510
RPL8	0.842	GAPDH	0.510
SRPR	0.921	RPL8	0.579
B2M	1.210	SRPR	0.588

The lower the M value for a gene, the more stable expression is across the samples

**Fig. 3:**
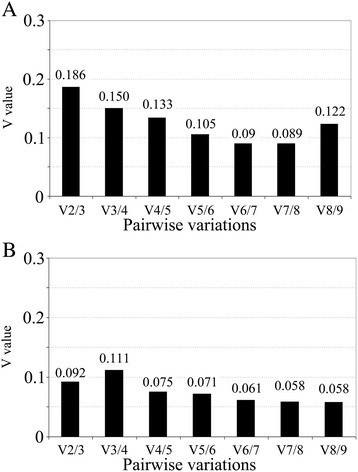
Pairwise variation plots for the 9 reference genes revealed the minimum number of reference genes required for normalization in: **a** canine OS primary tumors (minimum 4 genes) and **b** canine OS cell lines (minimum 2 genes)

Pairwise variation (V value), which reflects the optimal number of reference genes for normalization in tumors and cell lines, was also calculated. A lower the V value indicates lower variation between the selected combinations of reference genes. Normalization of gene expression data among 40 OS tumors required a minimum combination of 3 (V value is 0.15) and optimally 4 reference genes (V value <0.15), while a combination of 2 reference genes was sufficient for the OS cell lines (Fig. 3). These values were determined according to a cut-off V value of 0.15 as per published recommendations [21].

## Discussion

Selection of suitable reference genes is crucial for accurate interpretation of gene expression data [21, 22]. Many quality control measures, from initial sample collection to data analysis, should be evaluated critically prior to analysis of gene expression data [23, 24]. Reference genes, previously known as ‘housekeeping genes,’ are essential not only for normalizing the mRNA expression of target genes, but also for correcting variations in initial RNA sample input, extraction methods, and reaction efficiencies [25]. Failure to normalize gene expression data may result in inaccurate interpretation and promote false perception of target gene expression.

Numerous studies have been conducted to validate panels of reference genes in different tissues from different animals [26–29], including dogs. Previous studies on reference gene analysis using the GeNorm approach was done on soft tissues from dogs including skin, prostate, kidney, mammary gland, heart and liver tissues [19, 20]. Bone tissues are of mesenchymal origin and certainly have a set of genes expressed differentially compared to soft tissues. It is not known if the optimal reference genes would be the same as other soft tissues, hence this study was necessary. Besides that, there are only two other studies on reference genes on tumor specimens using the GeNorm analysis which are on canine soft tissue sarcoma (*n* = 6 tumors) [30] and canine mammary gland tumors (*n* = 22 tumors) [9]. Reference genes stably expressed in canine soft tissue sarcoma are β-Glucuronidase (GUSB) and proteasome subunit, beta type, 6 (PSMB6); while in canine mammary gland tumors were a combination of hypoxanthine-phosphoribosyl transferase, ATP-synthase subunit 5B, ribosomal protein L32 and ubiquitin. These two studies suggest different set of reference gene which are stably expressed as compared to the current study on canine osteosarcoma.

This study investigated the reliability of several reference genes expression in snap-frozen tumors and in cell lines of canine OS origin. The present study validated a panel of nine reference genes commonly used for qPCR investigations on dog tissues. Although this is not the first study to demonstrate the need for reference gene validation in tumor tissues from dogs, this is the first study to use OS tissues and to incorporate the largest number of snap-frozen canine tumor tissues and cell lines in a single canine reference gene validation study. The popular and established statistical tool geNorm (version 3.5) was used to calculate reference gene expression stability. For technical considerations, most ‘essential’ criteria outlined in the MIQE (Minimum Information for Publication of Quantitative Real-Time PCR Experiments) standards were employed in the current investigation in canine OS tissues [6]. The present study was unable to examine gene expression for biological replicates of OS tumors as recommended in the MIQE guidelines and power analysis was not conducted prior to the experiment to determine the number of samples necessary for valid conclusions, as the samples were obtained from naturally developed tumors in dogs and not from an experimental laboratory setting where the sample size can be controlled. The sample size in this study was based on sample availability, and with good quality RNA and sufficient RNA (quantity).

All nine reference genes tested in both canine OS snap-frozen tumors and cell lines showed acceptable expression stability with M values below 1.5. Overall, reference genes were much more stably expressed in cell lines (M values of 0.420–0.588) compared to those in tumor tissues (M values of 0.790–1.210), clearly indicating homogeneity among cell populations in cultured systems. In contrast, tumor tissues contain more heterogeneous cell populations.

Ribosomal protein genes (components of both 40S and 60S subunits) are highly expressed in various tissues and are preferred references for normalization in various models [8, 19, 20, 29], including in the present study of canine OS. Although there were slight differences in the ranking of genes (according to absolute M values) between those tested for the cell lines and tumors, *RPS5* was the most stable gene in both model systems. *RPS5* in combination with *RPS19* (for tumor tissues) or *HNRNPH* (for cell lines) showed the highest expression stability compared to other genes such as *B2M* and *GAPDH*, which are the most commonly used reference genes in many human and canine OS studies to date [10, 18, 31]. *GAPDH* expression did not appear to differ remarkably between OS samples, but its expression stability was much lower than the other reference genes investigated in the present study, which agrees with several previous reports [32, 33]. GAPDH is an enzyme involved in several metabolic pathways that are essential for cell growth and proliferation, and its expression has shown to differ in different tissue types and environment conditions [22, 34]. In an investigation of canine articular connective tissue, *GAPDH* and *B2M* were found to be highly stable [35], while in canine mammary tumors, *GAPDH* was less stable [9]. Furthermore, GAPDH protein expression in cultured cells may change depending upon cell density [34], and it was also found to be differentially expressed between tumors of epithelial origin and their normal counterparts [22]. Among canine OS tumors, *B2M* showed the lowest expression stability compared to the other eight candidate genes investigated in this study. Therefore, it is not recommended to rely on *B2M* nor *GAPDH* as a sole reference gene to normalize gene expression data.

Pairwise analysis of a combination of genes that can be used for normalization revealed that four reference genes for canine OS tumors and two for the cell lines were essential based on a recommended cut-off point. A lower V indicated smaller variation, suggesting that adding an additional gene did not significantly improve normalization. A cut-off value of 0.15 for pairwise variation is commonly used, indicating that the use of a set of reference genes with a pairwise variation results in valid normalization. As more genes are incorporated for normalization, the V value decreases to an optimal seven reference genes, which can be considered during normalization, given the expression data across canine OS tumors. When sample availability and RNA yield is limited, particularly from OS tumor materials, a minimum of three reference genes is acceptable, and four reference genes are optimal for normalization. OS typically shows a complex heterogeneous phenotype, and thus we recommend including multiple reference genes for the normalization of mRNA gene expression data.

The current study incorporated canine OS tumors, which are chemo-naive, and thus we cannot exclude the possibility of changes in reference gene stability in tumors induced by the various therapeutic modalities employed in clinical and experimental settings. If gene expression quantification comparing the effects of a given therapy is required, screening of a panel of reference genes may be essential prior to data normalization. Additionally, based on the assumption that RNA isolated from a specific tissue section represents the overall pooled expression in the tumor, RNA transcription in canine OS tumor tissues was quantified from a single tissue section from an individual OS tumor. Several other studies have recommended incorporating different parts of the same tumor to include separate biological replicates to more accurately quantify gene expression. However, this is often not feasible because of limited tissue availability. Further studies are necessary to test other potential or novel reference genes identified by global gene expression profiling methods and subsequently validated using other statistical algorithms. Because canine spontaneous OS is a clinically and biologically relevant model for human OS [36], we propose that multiple reference genes should be included in future normalization of gene expression data for both species to improve the accuracy and reliability of gene expression quantification.

## Conclusions

In conclusion, this study agreed with the consensus opinion that no single reference gene can accurately normalize given expression data. A combination of reference genes is recommended for normalizing the gene expression data from OS tumors and cell lines, with a preference for *RPS5* as a highly stable reference gene in canine OS.

## Abbreviations

B2M: β-2-Microglobulin
bp: base pair
CB: chondroblastic
cDNA: complementary DNA
FB: fibroblastic
GAPDH: Glyceraldehyde-3-phosphate dehydrogenase
GUSB: β-glucuronidase
HNRNPH: Heterogeneous nuclear ribonucleoprotein H
HPRT: Hypoxanthine guanine phosphoribosyl transferase
MIQE: Minimum Information for Publication of Quantitative Real-Time PCR Experiments
mRNA: messenger RNA
OB: osteoblastic
OS: osteosarcoma
PSMB6: Proteosome subunit beta type 6
qPCR: quantitative real-time PCR
RIN: RNA Integrity Number
RPL8: Ribosomal protein L8
RPS19: Ribosomal protein S19
RPS5: Ribosomal protein S5
SRPR: Signal recognition particle receptor
T_a_: annealing temperature
TL: telangiectic

## References

[1] Loukopoulos P, Robinson WF (2007). Clinicopathological relevance of tumour grading in canine osteosarcoma. J Comp Pathol.

[2] Kirpensteijn J, Kik M, Rutteman GR, Teske E (2002). Prognostic significance of a new histologic grading system for canine osteosarcoma. Vet Pathol.

[3] Selvarajah GT, Kirpensteijn J, van Wolferen ME, Rao NA, Fieten H, Mol JA (2009). Gene expression profiling of canine osteosarcoma reveals genes associated with short and long survival times. Mol Cancer.

[4] Paoloni M, Davis S, Lana S, Withrow S, Sangiorgi L, Picci P (2009). Canine tumor cross-species genomics uncovers targets linked to osteosarcoma progression. BMC Genomics.

[5] Drury S, Anderson H, Dowsett M (2009). Selection of REFERENCE genes for normalization of qRT-PCR data derived from FFPE breast tumors. Diagn Mol Pathol.

[6] Bustin SA, Benes V, Garson JA, Hellemans J, Huggett J, Kubiasta M (2009). The MIQE guidelines: minimum information for publication of quantitative real-time PCR experiments. Clin Chem.

[7] Derveaux S, Vandesompele J, Hellemans J (2010). How to do successful gene expression analysis using real-time PCR. Methods.

[8] Wood SH, Clements DN, McEwan NA, Nuttall T, Carter SD (2008). Reference genes for canine skin when using quantitative real-time PCR. Vet Immunol Immunopathol.

[9] Etschmann B, Wilcken B, Stoevesand K, von der Schulenburg A, Sterner-Kock A (2006). Selection of reference genes for quantitative real-time PCR analysis in canine mammary tumors using the GeNorm algorithm. Vet Pathol.

[10] Flint AF, U'Ren L, Legare ME, Withrow SJ, Dernell W, Hanneman WH (2004). Overexpression of the erbB-2 proto-oncogene in canine osteosarcoma cell lines and tumors. Vet Pathol.

[11] Fossey SL, Liao AT, McCleese JK, Bear MD, Lin J, Li PK (2009). Characterization of STAT3 activation and expression in canine and human osteosarcoma. BMC Cancer.

[12] De Maria R, Miretti S, Iussich S, Olivero M, Morello E, Bertotti A (2009). Met oncogene activation qualifies spontaneous canine osteosarcoma as a suitable pre-clinical model of human osteosarcoma. J Pathol.

[13] Takagi S, Kato Y, Asano K, Ohsaki T, Bosnakovski D, Hoshino Y (2005). Matrix metalloproteinase inhibitor RECK expression in canine tumors. J Vet Med Sci.

[14] Takagi S, Kitamura T, Hosaka Y, Ohsaki T, Bosnakovski D, Kadosawa T (2005). Molecular cloning of canine membrane-anchored inhibitor of matrix metalloproteinase, RECK. J Vet Med Sci.

[15] Shoieb AM, Hahn KA, Barnhill MA (1998). An in vivo/in vitro experimental model system for the study of human osteosarcoma: canine osteosarcoma cells (COS31) which retain osteoblastic and metastatic properties in nude mice. In Vivo.

[16] Barroga EF, Kadosawa T, Okumura M, Fujinaga T (1999). Establishment and characterization of the growth and pulmonary metastasis of a highly lung metastasizing cell line from canine osteosarcoma in nude mice. J Vet Med Sci.

[17] Kadosawa T, Nozaki K, Sasaki N, Takeuchi A (1994). Establishment and characterization of a new cell line from a canine osteosarcoma. J Vet Med Sci.

[18] Fieten H, Spee B, Ijzer J, Kik MJ, Penning LC, Kirpensteijn J (2009). Expression of hepatocyte growth factor and the proto-oncogenic receptor c-met in canine osteosarcoma. Vet Pathol.

[19] Brinkhof B, Spee B, Rothuizen J, Penning LC (2006). Development and evaluation of canine reference genes for accurate quantification of gene expression. Anal Biochem.

[20] Schlotter YM, Veenhof EZ, Brinkhof B, Rutten VP, Spee B, Willemse T (2009). A GeNorm algorithm-based selection of reference genes for quantitative real-time PCR in skin biopsies of healthy dogs and dogs with atopic dermatitis. Vet Immunol Immunopathol.

[21] Vandesompele J, De Preter K, Pattyn F, Poppe B, Van Roy N, De Paepa A (2002). Accurate Normalization of Real-Time Quantitative RT-PCR Data by Geometric Averaging of Multiple Internal Control Genes. Genome Biol.

[22] Rubie C, Kempf K, Hans J, Su T, Tilton B, Georg T (2005). Housekeeping gene variability in normal and cancerous colorectal, pancreatic, esophageal, gastric and hepatic tissues. Mol Cell Probes.

[23] Botling J, Edlund K, Segersten U, Tahmasebpoor S, Engstrom M, Sundstrom M (2009). Impact of thawing on RNA integrity and gene expression analysis in fresh frozen tissue. Diagn Mol Pathol.

[24] Becker C, Hammerle-Fickinger A, Riedmaier I, Pfaffl MW (2010). MRNA and microRNA quality control for RT-qPCR analysis. Methods.

[25] Peters IR, Peeters D (2007). Helps CR, day MJ. Development and application of multiple internal reference (housekeeper) gene assays for accurate normalisation of canine gene expression studies. Vet Immunol Immunopathol.

[26] Figueiredo MD, Salter CE, Andrietti AL, Vandenplas ML, Hurley DJ, Moore JN (2009). Validation of a reliable set of primer pairs for measuring gene expression by real-time quantitative RT-PCR in equine leukocytes. Vet Immunol Immunopathol.

[27] Nygard AB, Jorgensen CB, Cirera S, Fredholm M (2007). Selection of reference genes for gene expression studies in pig tissues using SYBR green qPCR. BMC Mol Biol.

[28] Olsvik PA, Softeland L, Lie KK (2008). Selection of reference genes for qRT-PCR examination of wild populations of Atlantic cod Gadus Morhua. BMC Res Notes.

[29] Penning LC, Vrieling HE, Brinkhof B, Riemers FM, Rothuizen J, Rutteman GR (2007). A validation of 10 feline reference genes for gene expression measurements in snap-frozen tissues. Vet Immunol Immunopathol.

[30] Zornhagen KW, Kristensen AT, Hansen AE, Oxboel J, Kjaer A (2015). Selection of suitable reference genes for normalization of genes of interest in canine soft tissue sarcomas using quantitative real-time polymerase chain reaction. Vet Comp Oncol.

[31] Miyajima N, Watanabe M, Ohashi E, Mochizuki M, Nishimura R, Ogawa H (2006). Relationship between retinoic acid receptor alpha gene expression and growth-inhibitory effect of all-trans retinoic acid on canine tumor cells. J Vet Intern Med.

[32] Lallemant B, Evrard A, Combescure C, Chapuis H, Chambon G, Raynal C (2009). Reference gene selection for head and neck squamous cell carcinoma gene expression studies. BMC Mol Biol.

[33] Nguewa PA, Agorreta J, Blanco D, Lozano MD, Gomez-Roman J, Sanchez BA (2008). Identification of importin 8 (IPO8) as the most accurate reference gene for the Clinicopathological analysis of lung specimens. BMC Mol Biol.

[34] Greer S, Honeywell R, Geletu M, Arulanandam R, Raptis L (2010). Housekeeping genes; expression levels may change with density of cultured cells. J Immunol Methods.

[35] Ayers D, Clements DN, Salway F, Day PJ (2007). Expression stability of commonly used reference genes in canine articular connective tissues. BMC Vet Res.

[36] Khanna C, Lindblad-Toh K, Vail D, London C, Bergman P, Barber L, et al. The Dog as a Cancer Model. Nat Biotechnol 2006;24:1065–6. 10.1038/nbt0906-1065b 16964204

